# Clinical Features and Outcomes of Necrotizing Pneumonia in a Peripheral Intensive Care Unit: A Case Series

**DOI:** 10.7759/cureus.99705

**Published:** 2025-12-20

**Authors:** Maria Beatriz Dias Vieira, Ana Rafaela Araújo, Daniela Alves, Margarida Midões Almeida, Eduardo Santos Ribeiro

**Affiliations:** 1 Intensive Care Unit, Unidade Local de Saúde da Região de Aveiro, Aveiro, PRT; 2 Internal Medicine, Unidade Local de Saúde da Região de Aveiro, Aveiro, PRT

**Keywords:** community-acquired pneumonia, intensive care unit, lung cavitation, necrotizing pneumonia, vv-ecmo

## Abstract

Necrotizing pneumonia (NP) is a rare but severe complication of community-acquired pneumonia (CAP). We describe three consecutive adult patients admitted to a peripheral intensive care unit (ICU) in Portugal between January 2023 and October 2024, all presenting with severe CAP complicated by bacteremia (*Klebsiella pneumoniae, *methicillin-sensitive* Staphylococcus aureus, *and *Streptococcus pneumoniae*). All patients required invasive mechanical ventilation, with Cases 2 and 3 also requiring vasopressor support shortly after ICU admission. NP was diagnosed by chest computed tomography (CT) on days 6, 6, and 11 of hospitalization, respectively, following unexpected clinical deterioration. Two patients developed refractory acute respiratory distress syndrome (ARDS) and were transferred to a tertiary center for venovenous extracorporeal membrane oxygenation (VV-ECMO) evaluation; both survived to hospital discharge. The third patient progressed to multiorgan failure and died on day 11. This case series illustrates the rapid clinical decline that can precede radiological confirmation of NP and highlights practical challenges faced in resource-limited settings, particularly regarding timely advanced imaging and referral. Further studies are needed to determine how these factors (timing of imaging, organ support availability, and ICU resource limitations) influence outcomes.

## Introduction

Necrotizing pneumonia (NP) is an uncommon but severe complication of community-acquired pneumonia (CAP), occurring in less than 1% of hospitalized CAP cases [1]. It is characterized by parenchymal necrosis with cavitation, micro-abscesses, and often concomitant bacteremia, and is associated with consistently high morbidity and mortality [2-4].

NP represents the most aggressive manifestation within a continuum that includes lung abscess and pulmonary gangrene [5]. It is defined by intense inflammation with consolidation, multifocal parenchymal necrosis, and numerous small cavitary lesions resulting from progressive destruction of the bronchovascular architecture [6]. This vascular compromise creates poorly perfused regions with limited antibiotic penetration, permitting uncontrolled infection and promoting further tissue devitalization [5]. Pathogenesis may involve aspiration, typically associated with polymicrobial anaerobic infections, direct invasion by highly virulent organisms, such as *Staphylococcus aureus* or *Klebsiella pneumoniae*, or septic embolization in the context of bacteremia or right-sided endocarditis [3,4]. While a lung abscess usually presents as a single circumscribed purulent cavity, NP is characterized by multifocal necrosis, and pulmonary gangrene represents the extreme end of this continuum, marked by extensive parenchymal destruction and sloughing of lung segments or entire lobes [5].

Management of NP remains challenging, largely because formal guidelines are lacking and available evidence stems mainly from case reports and retrospective studies [5]. Early diagnosis is difficult, as initial imaging often resembles severe CAP, and chest computed tomography (CT) is essential for identifying cavitation, micro-abscesses, and parenchymal necrosis [4,7]. Treatment is not standardized and relies on the prompt initiation of broad-spectrum antibiotics, aggressive supportive care, and drainage of associated complications. In cases complicated by refractory acute respiratory distress syndrome (ARDS), extracorporeal membrane oxygenation (ECMO) may be required [5,8,9]. Although most patients are managed medically, surgical intervention may become necessary when there is ongoing deterioration despite optimal therapy [2]. Mortality remains substantial, particularly among patients with septic shock, multiorgan failure, or those requiring advanced rescue modalities [5]. These challenges are further amplified in peripheral or resource-limited hospitals, where delays in interventional procedures or transfer for ECMO may adversely impact prognosis [7].

This case series describes three adult patients with NP admitted to the intensive care unit (ICU) of a peripheral Portuguese hospital, highlighting diagnostic challenges, rapid clinical deterioration, the need for advanced organ support, and the multidisciplinary complexity involved in management. The primary aim was to characterize the clinical course, timing of imaging, and organ support requirements of NP in a setting without on-site ECMO, interventional radiology, or thoracic surgery. By doing so, this study seeks to address a gap in contemporary adult NP literature and provide insight into the practical challenges of managing this severe condition in resource-limited ICUs, particularly when comparing outcomes with large tertiary-center cohorts where diagnostic and therapeutic resources differ substantially.

This retrospective descriptive study was conducted in a 14-bed mixed medical-surgical ICU at a peripheral district hospital in Portugal, located approximately 45 minutes by road from the nearest tertiary referral center. Chest CT was available on-site but subject to scheduling constraints outside daytime hours.

All consecutive adult patients (≥18 years) admitted to the ICU between January 2023 and October 2024 with severe community-acquired pneumonia who subsequently developed unexpected clinical deterioration were screened. Repeat chest CT imaging was performed in these cases, and necrotizing pneumonia was diagnosed based on radiologic criteria, including the presence of multiple cavitations, areas of non-enhancing lung parenchyma, micro-abscesses, or liquefactive necrosis within consolidated lung tissue, in accordance with previously described radiologic definitions. Only patients with CT-confirmed parenchymal necrosis were included in this case series. All NP diagnoses were based on formal radiology reports and independently reviewed by the treating intensivist team.

Clinical, laboratory, microbiological, and imaging data were retrospectively extracted from electronic medical records to characterize patient presentation, timing of imaging, organ support requirements, and in-hospital clinical course.

Prespecified outcomes included ICU mortality, need for invasive mechanical ventilation, vasopressor support, development of multiorgan dysfunction, requirement for renal replacement therapy, and referral for ECMO evaluation. Length of ICU stay and total hospital length of stay were also recorded.

Microbiological evaluation included blood cultures obtained at ICU admission and during episodes of clinical deterioration, as well as respiratory samples (sputum or endotracheal aspirates) collected prior to antimicrobial escalation whenever feasible. Pathogen identification and antimicrobial susceptibility testing were performed in accordance with European Committee on Antimicrobial Susceptibility Testing (EUCAST) standards. Multiplex respiratory polymerase chain reaction (PCR) testing and specific virulence factor assays were not routinely available at our institution and were therefore not performed.

Regarding operational definitions for key entities in this case series, ARDS was defined according to the Berlin definition (originally published in 2012 and subsequently refined in 2023-2024), including acute onset of respiratory failure within one week of a known clinical insult, bilateral pulmonary opacities on chest imaging not fully explained by cardiac failure or fluid overload, and impaired oxygenation requiring invasive mechanical ventilation. Septic shock was defined according to the Sepsis-3 criteria, as sepsis with persistent hypotension requiring vasopressor support to maintain a mean arterial pressure ≥65 mmHg and serum lactate >2 mmol/L despite adequate fluid resuscitation. Acute kidney injury (AKI) was classified using the Acute Kidney Injury Network (AKIN) criteria based on changes in serum creatinine and/or urine output (Stage 1: serum creatinine increase ≥0.3 mg/dL within 48 hours or ≥1.5 times baseline within 7 days, or urine output <0.5 mL/kg/h for >6 hours; higher stages reflect greater creatinine increases or more prolonged oliguria, with Stage 3 including any patient requiring renal replacement therapy). AKIN staging was retrospectively mapped to Kidney Disease: Improving Global Outcomes (KDIGO) definitions, as these classifications are considered largely equivalent in contemporary clinical practice.

Part of this work was previously presented in abstract form at the 38th ESICM Annual Congress - LIVES 2025 in Munich, Germany, and published as a conference abstract in Intensive Care Medicine Experimental (2025; 13 (Suppl 1):000173). The present article constitutes the first complete and original report of these cases.

## Case presentation

Case 1

**Figure 1:**
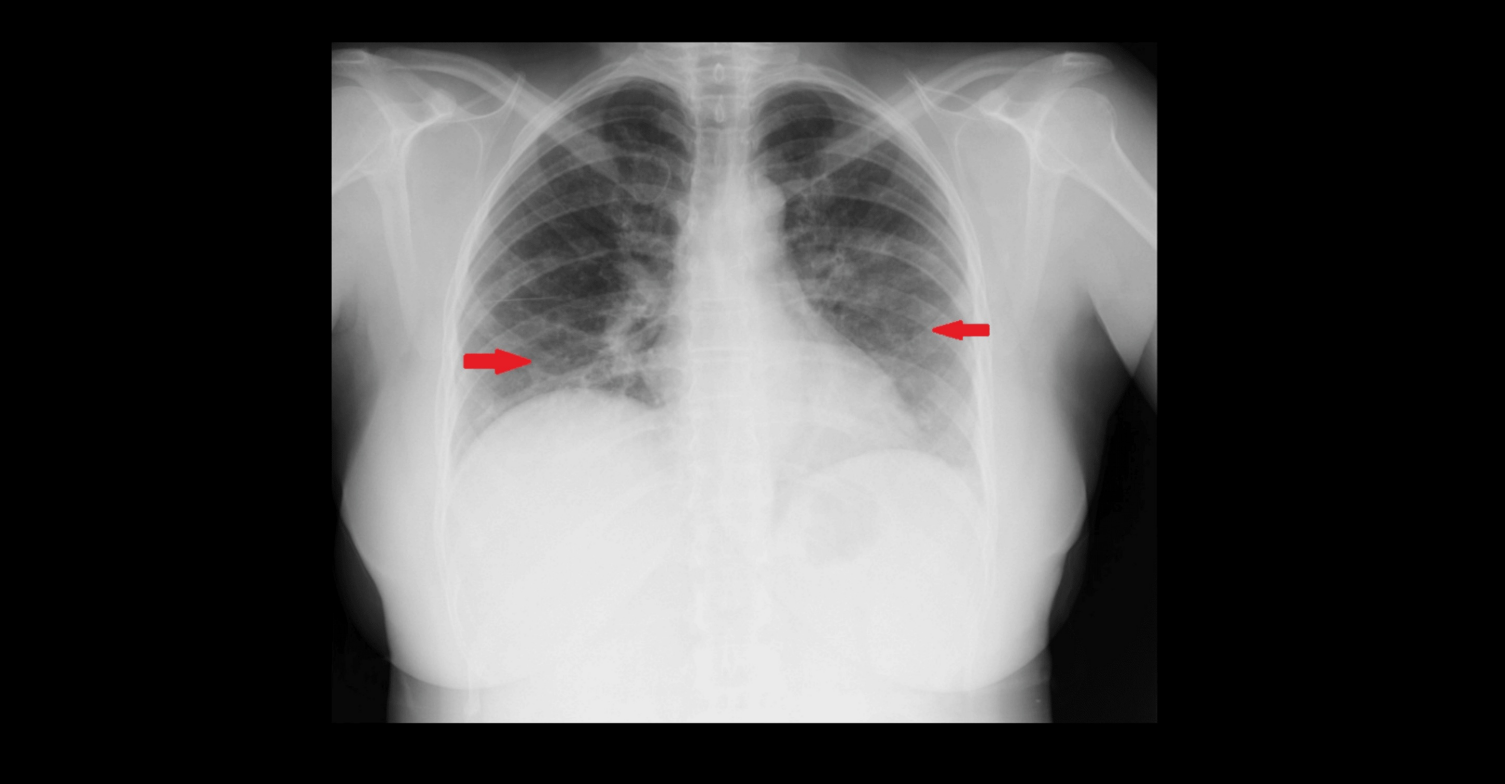
Chest radiograph on hospital admission showing diffuse bilateral pulmonary infiltrates (arrows), consistent with community-acquired pneumonia No cavitation or necrotizing features are evident at this stage.

We present the case of a 45-year-old woman with hypertension treated with atenolol, who was previously independent in daily activities. She presented to the emergency department with a one-week history of chest pain associated with dry cough, nocturnal fever up to 38.5 °C, nausea, and malaise. On admission, she was febrile (39.2 °C), tachycardic (127 bpm), tachypneic, and had bilateral pulmonary crackles. Laboratory testing revealed leukocytosis, elevated C-reactive protein, and procalcitonin (Table 1). Chest radiography demonstrated diffuse bilateral infiltrates (Figure 1).

**Table 1:** Admission laboratory values for Case 1 AST: aspartate aminotransferase; CRP: C-reactive protein; GGT: gamma-glutamyl transferase; INR: international normalized ratio; WBC: white blood cell count

Parameter	Result	Reference range
Hemoglobin	10.6 g/dL	13.0–18.0 g/dL
WBC	16.2 ×10⁹/L	4.1–11.1 ×10⁹/L
Neutrophils	13.35 ×10⁹/L	1.8–7.5 ×10⁹/L
Platelets	178 ×10⁹/L	150–500 ×10⁹/L
INR	1.6	0.7–1.3
D-dimer	5027 ng/mL	<500 ng/mL
Creatinine	1.16 mg/dL	0.70–1.30 mg/dL
Potassium	3.4 mmol/L	3.5–5.5 mmol/L
AST	130 U/L	13–38 U/L
GGT	48 U/L	10–60 U/L
CRP	33.17 mg/dL	<0.5 mg/dL
Procalcitonin	4.53 ng/mL	<0.1 ng/mL

At admission, the patient had a CURB-65 score of 2 and a Pneumonia Severity Index (PSI) of Class IV, indicating severe community-acquired pneumonia (CAP) requiring early intensive care. Testing for viral pathogens (SARS-CoV-2, RSV, influenza A and B, adenovirus) as well as atypical agents, including* Mycoplasma pneumoniae* PCR and Legionella urinary antigen, was carried out and returned negative results. She was started on amoxicillin/clavulanate plus azithromycin, and, given the severity of respiratory failure, was immediately admitted to the ICU for close monitoring and advanced respiratory support.

Within the first 24 hours, she developed worsening tachypnea and hypoxemia, requiring oxygen supplementation and subsequently non-invasive ventilation. Due to further clinical deterioration, antimicrobial therapy was escalated to piperacillin-tazobactam. Sputum, blood, and urine cultures were collected.

Despite supportive measures, the patient exhibited progressive respiratory failure, ultimately requiring orotracheal intubation and invasive mechanical ventilation. Blood and sputum cultures grew *Klebsiella pneumoniae*, supporting the diagnosis of severe CAP complicated by bacteremia. The isolate was fully susceptible to standard antibiotics. Specific testing for hypervirulent phenotypes (including string test, hypermucoviscosity assessment, or genotypic markers) is not performed in our laboratory and was therefore not available.

Over the following days, she developed persistent fever, rising inflammatory markers, and increasing ventilatory requirements consistent with worsening systemic inflammatory response. New microbiological samples were collected, and antimicrobial therapy was escalated to ceftazidime plus linezolid.

**Figure 2:**
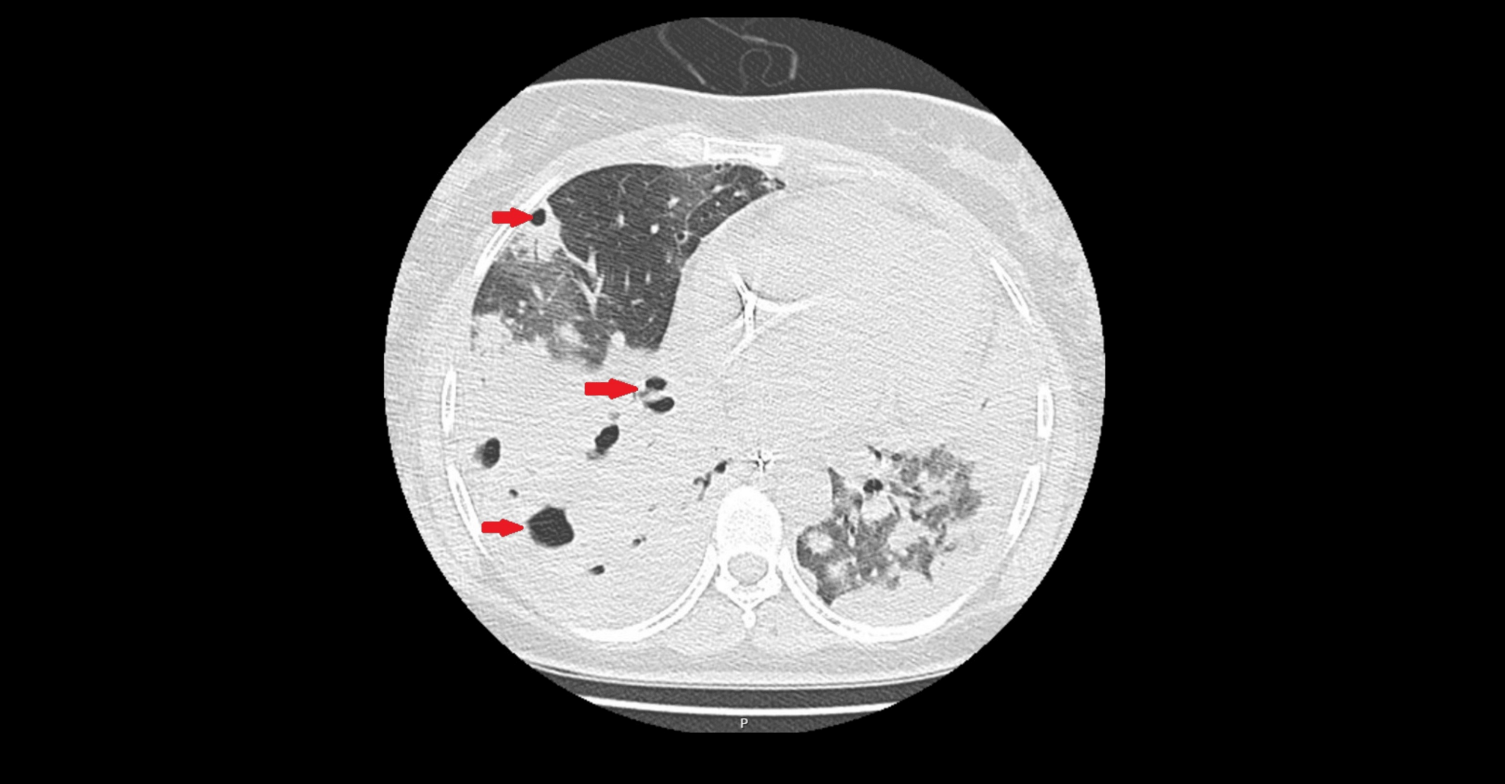
Chest CT obtained on ICU day 6 demonstrates extensive areas of non-enhancing lung parenchyma with multiple cavitated lesions (arrows), consistent with necrotizing pneumonia CT: computed tomography; ICU: intensive care unit

On day 6 of ICU stay, due to abrupt respiratory decompensation and escalating inflammatory biomarkers, a chest CT was obtained and revealed extensive bilateral parenchymal necrosis with multiple cavitated lesions (Figure 2), establishing the diagnosis of necrotizing pneumonia (NP).

Following the diagnosis of NP, the patient progressed to severe ARDS, refractory to protective ventilation and prone positioning. On day 7, she was transferred to a tertiary center for venovenous extracorporeal membrane oxygenation (VV-ECMO) rescue. She underwent tracheostomy on day 11 of her tertiary hospital stay and remained tracheostomized for 30 days. She stayed on VV-ECMO for 29 days, with a total ICU stay of 38 days at the tertiary center. She was subsequently returned to the referring hospital for 10 additional days, completing a total hospital stay of 55 days. She ultimately survived, was discharged home, and had residual muscular weakness at discharge.

Case 2

**Figure 3:**
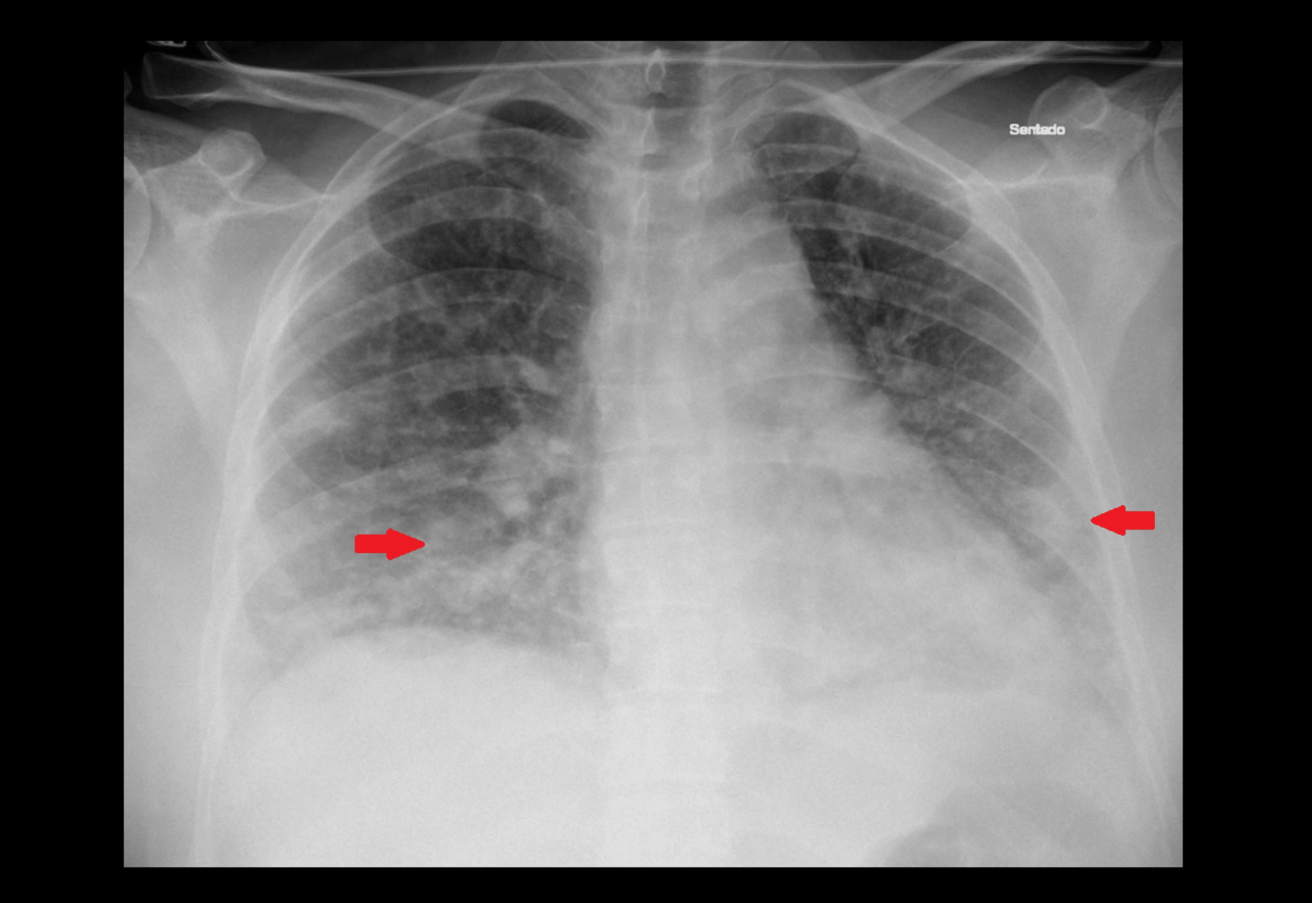
Chest radiograph on hospital admission showing bilateral pulmonary infiltrates (arrows), consistent with community-acquired pneumonia prior to the development of necrotizing changes

We present the case of a 39-year-old woman, previously independent, with a history of heterozygous Factor V Leiden mutation and recurrent deep-vein thrombosis under chronic apixaban therapy. She presented to the emergency department with a five-day history of pleuritic back pain, dry cough, and progressive dyspnea. On arrival, she appeared acutely ill, hypotensive (74/32 mmHg), tachycardic (138 bpm), tachypneic (45 breaths/min), and hypoxemic (oxygen saturation (SpO₂) 92% on 3 L/min O₂). Arterial blood gas analysis showed severe type 1 respiratory failure (partial pressure of oxygen (PaO₂) 55 mmHg) and elevated lactate (3.3 mmol/L). The chest radiography revealed bilateral infiltrates (Figure 3). Laboratory evaluation (Table 2) demonstrated acute kidney injury, hepatic dysfunction, and markedly raised inflammatory biomarkers.

**Table 2:** Admission laboratory values for Case 2. ALT: alanine aminotransferase; AST: aspartate aminotransferase; CRP: C-reactive protein; GGT: gamma-glutamyl transferase; INR: international normalized ratio; LDH: lactate dehydrogenase; WBC: white blood cell count

Parameter	Result	Reference range
Hemoglobin	13.1 g/dL	13.0–18.0 g/dL
WBC	7.7 ×10⁹/L	4.1–11.1 ×10⁹/L
Platelets	205 ×10⁹/L	150–500 ×10⁹/L
INR	1.9	0.7–1.3
Urea	78.1 mg/dL	19–51 mg/dL
Creatinine	1.85 mg/dL	0.70–1.30 mg/dL
Sodium	133 mmol/L	132–146 mmol/L
Troponin I	253.4 pg/mL	<47.3 pg/mL
AST	64 U/L	13–38 U/L
ALT	54 U/L	10–49 U/L
LDH	328 U/L	46–171 U/L
Total bilirubin	2.83 mg/dL	0.3–1.2 mg/dL
Direct bilirubin	1.8 mg/dL	<0.3 mg/dL
Alkaline phosphatase	100 U/L	46–116 U/L
GGT	58 U/L	10–60 U/L
CRP	51.89 mg/dL	<0.5 mg/dL
Procalcitonin	7.42 ng/mL	<0.1 ng/mL

At admission, the patient had a CURB-65 score of 3 (tachypnea, hypotension, elevated urea) and a PSI score of 144 (Class IV), indicating severe pneumonia with a high risk of adverse outcomes and supporting the need for early ICU-level care.

**Figure 4:**
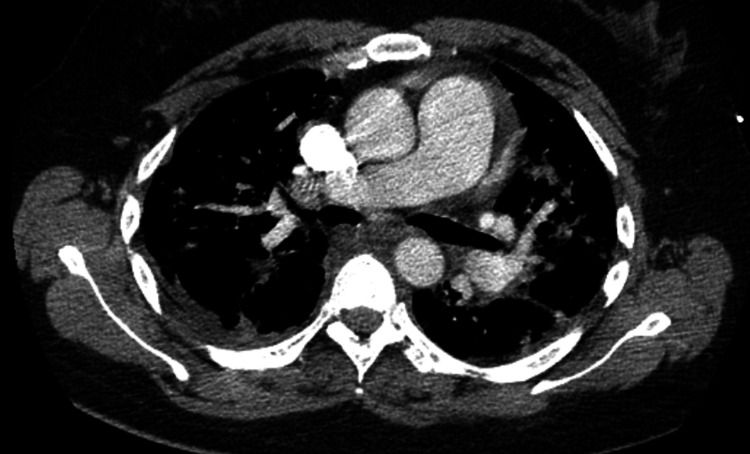
Chest CT angiogram performed on hospital admission showing no evidence of pulmonary embolism CT: computed tomography

**Figure 5:**
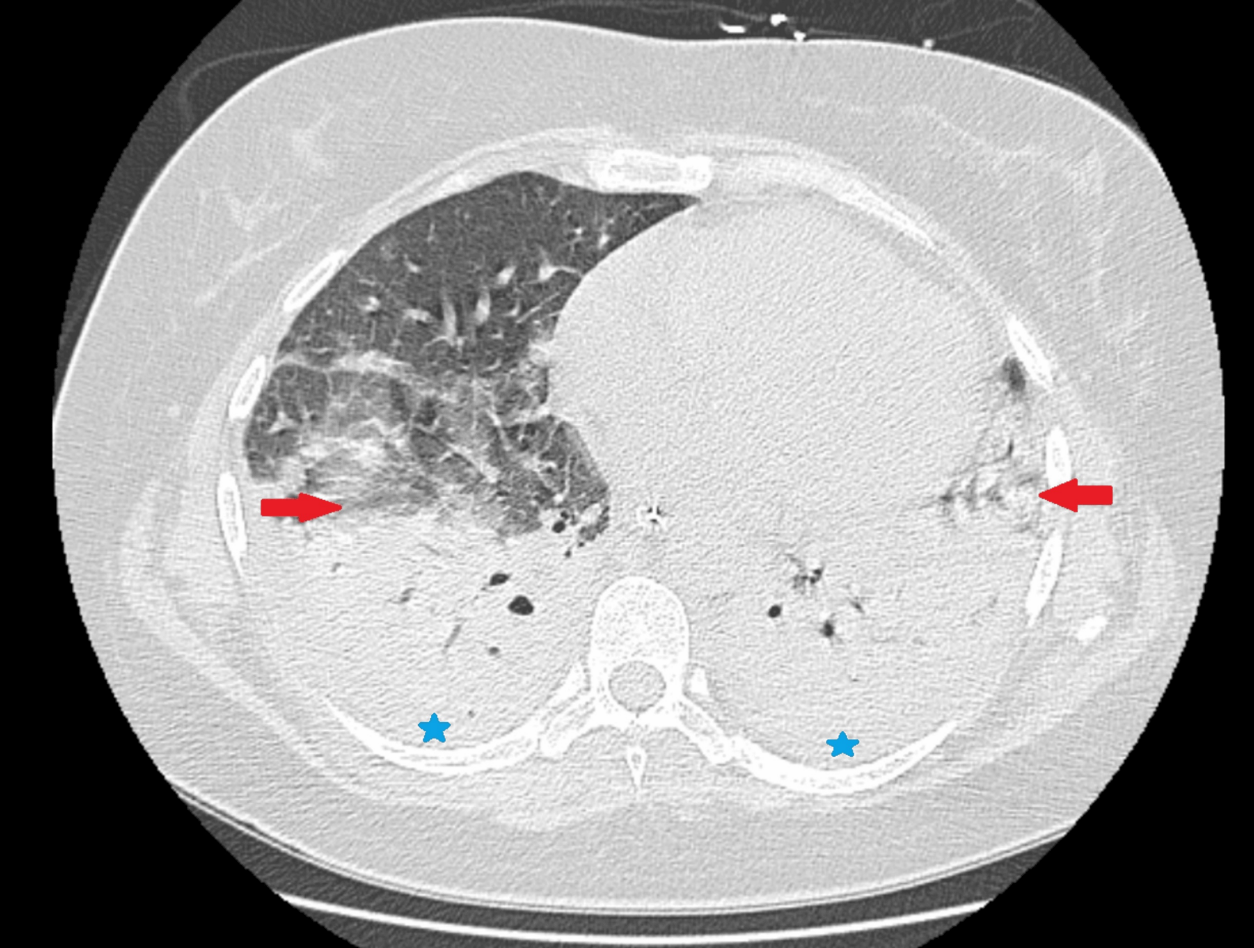
Chest CT on hospital admission demonstrating bilateral ground-glass opacities with focal consolidations (arrows) and small pleural effusions (stars), without radiological features of necrotizing pneumonia at this stage CT: computed tomography

A chest CT angiogram (Figure 4) excluded pulmonary embolism but revealed bilateral ground-glass opacities with small consolidations and pleural effusions (Figure 5), consistent with severe CAP. Empiric ceftriaxone plus azithromycin was initiated after obtaining blood and respiratory cultures. Viral (SARS-CoV-2, RSV, influenza A and B, adenovirus) and atypical pathogen testing, including *Mycoplasma pneumoniae* PCR and Legionella urinary antigen, was performed and yielded negative results.

Due to progressive respiratory distress and persistent hypotension, the patient was admitted to the ICU, where she required endotracheal intubation, invasive mechanical ventilation, and vasopressor support. Blood and sputum cultures later grew methicillin-sensitive *Staphylococcus aureus* (MSSA), prompting escalation of antibiotic therapy to intravenous flucloxacillin. The isolate was methicillin-sensitive with a fully susceptible antibiogram; nonetheless, Panton-Valentine Leucocidin (PVL) detection and broader toxin profiling are not routinely performed in our laboratory and were therefore not obtained.

**Figure 6:**
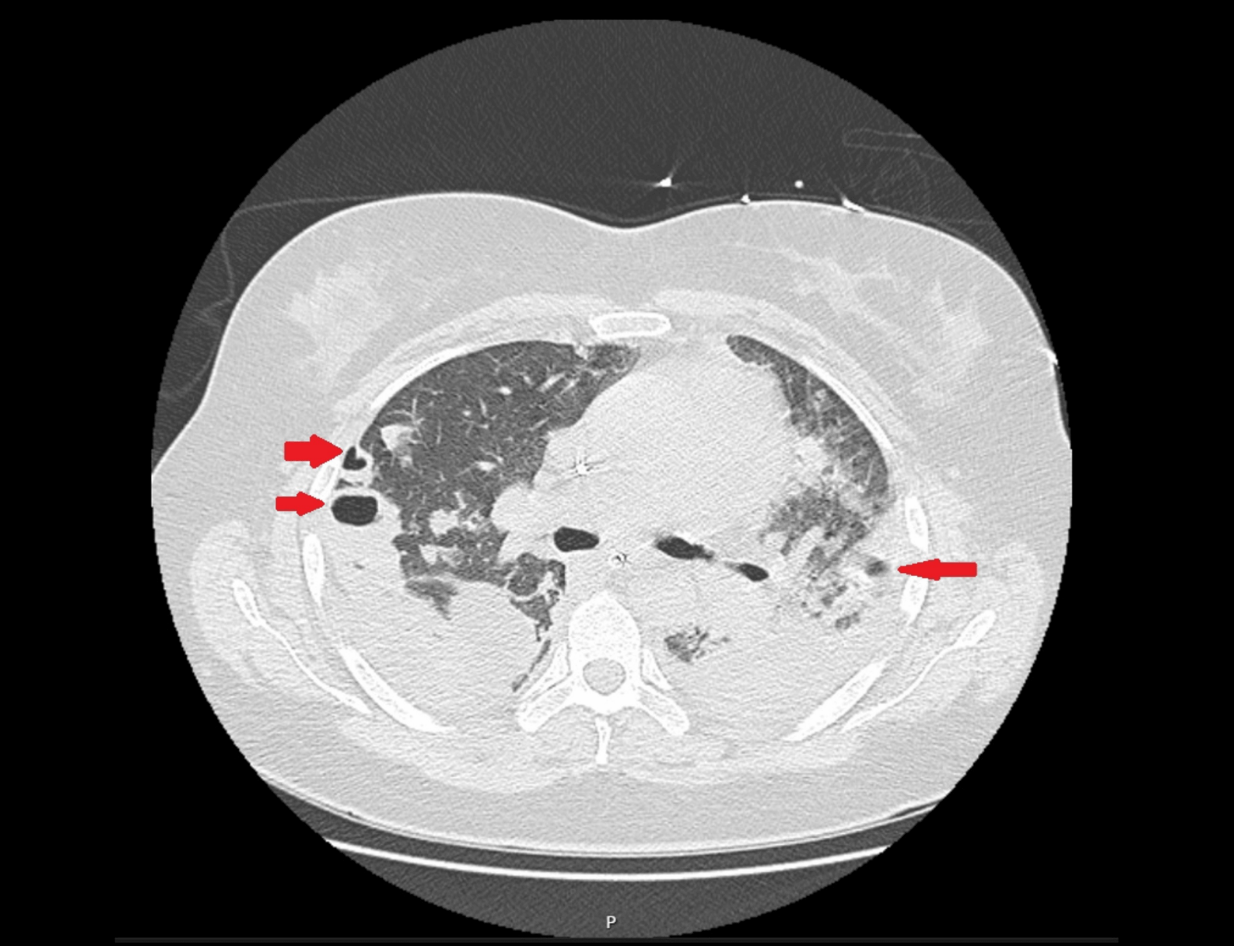
Repeat chest CT obtained on ICU day 6 demonstrating bilateral parenchymal non-enhancement, multiple cavitated nodules, and evolving pulmonary abscesses (arrows), consistent with necrotizing pneumonia CT: computed tomography; ICU: intensive care unit

Despite targeted antimicrobial therapy, the patient developed worsening respiratory failure and multiorgan dysfunction, including acute kidney injury (AKIN 2), hepatic impairment, and coagulopathy. On day 6 of hospitalization, in the setting of abrupt respiratory deterioration, persistent fever, and rising inflammatory biomarkers, a repeat chest CT demonstrated extensive bilateral parenchymal necrosis with multiple cavitated nodules and pulmonary abscesses, establishing the diagnosis of NP (Figure 6). The scan also showed a small left pleural empyema and a right-sided bronchopleural fistula.

Her oxygenation continued to decline, and she underwent a trial of prone positioning with no significant improvement. Given the severity of hypoxemia and the rapid progression of NP, she was subsequently transferred to a tertiary cardiothoracic center for VV-ECMO consideration. She ultimately improved without requiring ECMO, and the bronchopleural fistula did not necessitate surgical intervention following cardiothoracic surgical evaluation. She spent 15 days in the tertiary center ICU and was subsequently returned to the referring hospital for 12 additional days, for a total hospital stay of 33 days. She survived, was discharged home, and had residual muscular weakness and anxiety at discharge.

Case 3

We present the case of a 61-year-old man, previously autonomous, with a history of long-term smoking (36 pack-years), hypertension, dyslipidemia, peripheral arterial disease, hepatic steatosis, and chronic daily alcohol consumption. He presented to the emergency department with a five-day history of productive cough, progressive dyspnea, and three days of fever up to 39 °C. On arrival, he appeared acutely unwell, febrile (38.7 °C), tachycardic (110 bpm), hypotensive (99/57 mmHg), and tachypneic (>30 breaths/min), with oxygen saturation of 94% on 4 L/min O₂. Arterial blood gas analysis revealed type 1 respiratory failure (PaO₂/fraction of oxygen in the inspired air (FiO₂)) 205) and hyperlactatemia (3.3 mmol/L). Laboratory evaluation demonstrated leukopenia, lymphopenia, thrombocytopenia, acute kidney injury AKIN stage 2 (creatinine baseline 0.69 mg/dL), markedly elevated urea, hepatic cytolysis, and elevated C-reactive protein (Table 3).

**Table 3:** Admission laboratory values for Case 3 ALT: alanine aminotransferase; AST: aspartate aminotransferase; CRP: C-reactive protein; LDH: lactate dehydrogenase; WBC: white blood cell count

Parameter	Result	Reference range
Hemoglobin	11.6 g/dL	13.0–18.0 g/dL
WBC	3.8 ×10⁹/L	4.1–11.1 ×10⁹/L
Neutrophils	2.93 ×10⁹/L	1.8–7.5 ×10⁹/L
Lymphocytes	0.22 ×10⁹/L	1.1–3.5 ×10⁹/L
Platelets	108 ×10⁹/L	150–500 ×10⁹/L
Urea	127.2 mg/dL	19–51 mg/dL
Creatinine	1.74 mg/dL	0.70–1.30 mg/dL
Sodium	138.6 mmol/L	132–146 mmol/L
Potassium	3.06 mmol/L	3.5–5.5 mmol/L
AST	162 U/L	13–38 U/L
ALT	80 U/L	10–49 U/L
LDH	414 U/L	46–171 U/L
CRP	36.24 mg/dL	<0.5 mg/dL

Based on his presentation, he had a CURB-65 score of 3 (urea >7 mmol/L, respiratory rate ≥30/min, hypotension) and a PSI of 143 (Class IV), indicating severe CAP with high predicted mortality.

**Figure 7:**
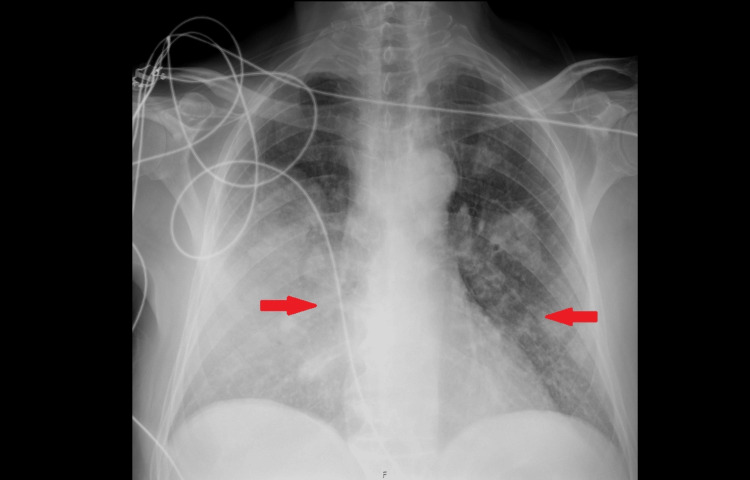
Chest radiograph on hospital admission showing bilateral pulmonary infiltrates (arrows), consistent with severe community-acquired pneumonia without cavitation

**Figure 8:**
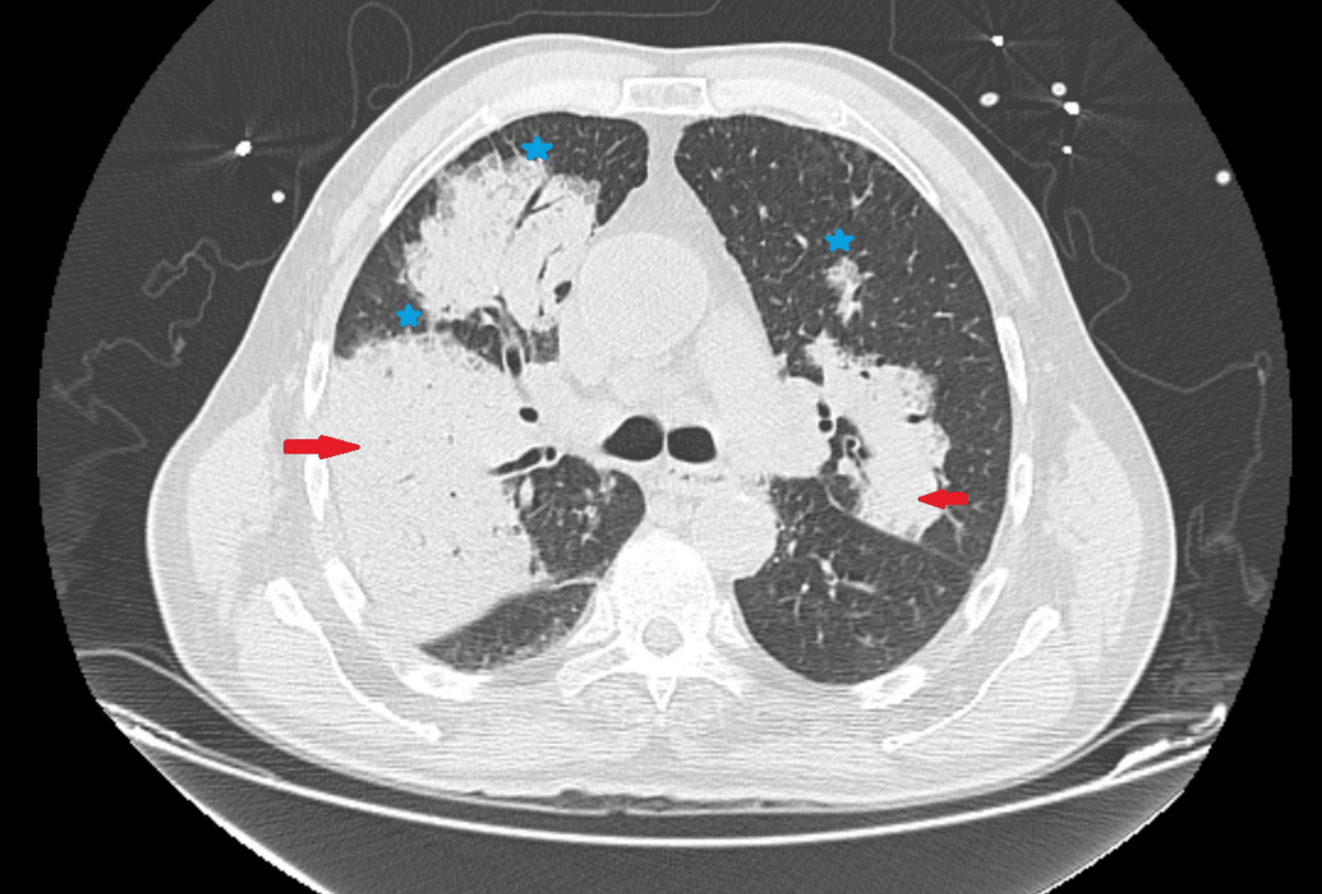
Chest CT obtained on hospital admission demonstrating multifocal bilateral ground-glass opacities (stars) and areas of consolidation (arrows), without radiological evidence of necrosis or cavitation CT: computed tomography

Chest radiography showed bilateral infiltrates (Figure 7), and a chest CT confirmed multifocal bilateral infiltrates with ground-glass opacities, micronodularity, and areas of consolidation (Figure 8). Viral testing (SARS-CoV-2, RSV, influenza A and B, adenovirus), along with atypical pathogen screening, including *Mycoplasma pneumoniae* PCR and Legionella urinary antigen, was performed and was negative. He was then started on high-flow nasal oxygen, ceftriaxone, and azithromycin. Microbiological workup revealed blood cultures positive for *Streptococcus pneumoniae*, and antimicrobial therapy was continued along with penicillin G. The *Streptococcus pneumoniae* isolate was susceptible to penicillin and other first-line agents; however, serotyping and extended susceptibility testing beyond the routine antibiogram are not available in our center and were not performed. Due to persistent respiratory distress and hemodynamic instability, he was admitted to the ICU.

Over the following days, he exhibited progressive multiorgan dysfunction, including worsening renal injury (progression to AKIN stage 3), rising lactate, thrombocytopenia, and hepatic dysfunction. Persistent fever, escalating inflammatory markers, and refractory hypoxemia prompted the collection of new microbiological samples and the escalation of antimicrobial therapy to meropenem plus linezolid from days 10 to 11.

**Figure 9:**
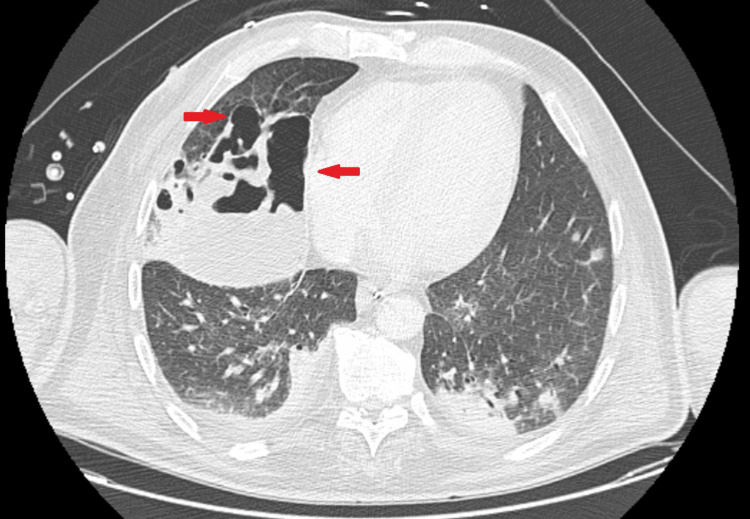
Repeat chest CT performed on ICU day 11 showing extensive bilateral liquefactive parenchymal necrosis with multiple cavitated lesions and areas of non-enhancement (arrows), confirming advanced necrotizing pneumonia and marked radiological progression from admission CT: computed tomography. ICU: intensive care unit

A chest CT performed revealed extensive bilateral parenchymal necrosis with multiple cavitated lesions, confirming NP and demonstrating marked radiological progression from admission (Figure 9). The patient’s condition worsened further on day 11, with severe anuria, rapidly rising creatine kinase consistent with rhabdomyolysis, and refractory metabolic acidosis.

Given the severity of shock, anuria, rhabdomyolysis (myoglobin 36,000 ng/mL), and metabolic derangement, continuous renal replacement therapy (CRRT) was initiated immediately. The circuit incorporated a Jafron HA380 hemoadsorption cartridge (Jafron Biomedical Co., Ltd., Zhuhai City, China), aiming to enhance cytokine and myoglobin clearance in the context of fulminant septic shock and rhabdomyolysis. Despite CRRT with hemoadsorption, metabolic acidosis persisted, and organ failure continued to progress.

Due to worsening hypoxemia, escalating norepinephrine requirements, and severe multiorgan dysfunction necessitating CRRT, along with aggravated rhabdomyolysis (myoglobin 41,000 ng/mL), the patient was deemed ineligible for ECMO rescue. He evolved to refractory shock and died later the same day.

Table 4 summarizes the clinical findings from the three patients admitted to our ICU.

**Table 4:** Summary of clinical characteristics, diagnostic timelines, organ support requirements, and outcomes of the three patients with necrotizing pneumonia included in this case series. The table highlights time to CT, types of organ dysfunction, need for advanced respiratory support, hospital and ICU LOS, and variability in prognosis across cases. AKI: acute kidney injury; AKIN: Acute Kidney Injury Network classification; APACHE II: Acute Physiology and Chronic Health Evaluation II score; CT: computed tomography; DVT: deep vein thrombosis; LOS: length of stay; MSSA: *methicillin-sensitive Staphylococcus aureus;* PAD: peripheral arterial disease; SAPS II: Simplified Acute Physiology Score II; SOFA: Sequential Organ Failure Assessment score; VV-ECMO: veno-venous extracorporeal membrane oxygenation * Decisions about antibiotic therapy were based on clinical and imaging evaluation, in agreement with the antimicrobial stewardship team, and only the days of therapy administered in the peripheral ICU were recorded.

Parameter	Case 1	Case 2	Case 3
Age / Sex	45 / Female	39 / Female	61 / Male
Comorbidities	Hypertension	Factor V Leiden mutation, recurrent DVT	Hypertension, dyslipidemia, PAD, smoking, hepatic steatosis, chronic alcohol
Pathogen	Klebsiella pneumoniae	MSSA	Streptococcus pneumoniae
SOFA at admission / APACHE II / SAPS II	11 / 23 / 35	13 / 28 / 50	9 / 17 / 40
Symptom-to-admission (days)	7	5	5
Admission-to-CT (days)	6	6	11
Admission-to-intubation (days)	1	0	1
Need for vasopressors	No	Yes	Yes
AKI stage	None	AKIN 2	AKIN 3
Antibiotics*	3 days of amoxicillin/clavulanate plus azithromycin; 4 days of piperacillin-tazobactam; less than a day of ceftazidime plus linezolid	2 days of ceftriaxone plus azithromycin; 4 days of flucloxacillin	3 days of ceftriaxone plus azithromycin; 7 days of penicillin G; 1 day of meropenem plus linezolid
Prone positioning	Yes	Yes	No
ECMO candidacy / implementation	29 days under VV-ECMO	Transferred for VV-ECMO consideration but improved without it	Not eligible
Duration of mechanical ventilation (days)	47	16	10
Tracheostomy	Yes (30 days)	No	No
Peripheral ICU length of stay (days)	7 (transferred)	6 (transferred)	11 (died)
Tertiary center ICU length of stay (days)	38	15	-
Return to peripheral hospital / length of stay (days)	Yes / 10	Yes / 12	-
Hospital total length of stay (days)	55	33	11 (died)
Final outcome	Survived	Survived	Died
Disposition at hospital discharge	Home	Home	-
Major sequelae at discharge	Muscular weakness	Muscular weakness, anxiety	-

## Discussion

Necrotizing pneumonia (NP) remains a rare but highly destructive complication of CAP, characterized by parenchymal necrosis, cavitation, and microabscess formation [3,4]. The three patients presented in this series demonstrated the classic features described in the literature, including abrupt respiratory deterioration, marked systemic inflammation, and rapid progression to multiorgan dysfunction. These manifestations reflect the underlying pathophysiology of extensive bronchovascular destruction, which promotes tissue devitalization and limits antibiotic penetration [4,5].

Regarding the microbiological patterns observed in our cases (*Staphylococcus aureus, Klebsiella pneumoniae, *and *Streptococcus pneumoniae*), they’re consistent with the main etiologic agents reported in adult NP [3,4]. *Staphylococcus* *aureus*, identified in Case 2, remains one of the most frequent causes of fulminant necrotizing infections and pulmonary gangrene, particularly when toxin-producing strains are involved [4]. *Klebsiella* *pneumoniae*, isolated in Case 1, is similarly associated with severe cavitary disease, bacteremia, and rapid tissue necrosis. Pneumococcal NP, as in Case 3, has been increasingly recognized in contemporary series, including the recent multicenter study by Boppana et al., where it accounted for a significant proportion of monomicrobial infections [1]. The absence of PVL and hypervirulent phenotype testing in our cases limits direct comparison with some international series, and this should be considered when interpreting our findings.

Early diagnosis of NP remains challenging, as initial chest radiography frequently resembles severe community-acquired pneumonia, with definitive features, such as cavitation, areas of non-enhancement, or multiple small abscesses, becoming apparent only on chest CT [4]. In our series, CT confirmation occurred on days 6, 6, and 11 after admission, a timeframe consistent with published data from non-ECMO-capable centers, where median times to radiological confirmation of approximately 6 to 7 days have been reported and are therefore not considered unusually late [1]. Nevertheless, prolonged intervals to definitive imaging have been recurrently described in reports from Portuguese peripheral hospitals, reflecting structural and organizational constraints rather than deviations from accepted clinical practice [2].

Although delayed CT has historically been viewed as a potential contributor to adverse outcomes, available evidence does not demonstrate a direct association between the timing of CT confirmation and the subsequent need for rescue therapies such as ECMO or surgical intervention. Instead, outcomes appear to be more strongly driven by disease severity and the requirement for invasive mechanical ventilation, with surgical management rarely successful and ECMO reserved for selected patients with potentially reversible respiratory failure [1,10]. Similarly, mortality and prolonged length of stay have been linked to overall illness severity rather than imaging timing, with necrotizing changes predicting longer hospitalization but not increased mortality [1]. Consequently, evidence supporting earlier CT as a means to improve outcomes or reduce the need for rescue therapies remains limited, underscoring the need for further investigation into the clinical impact of diagnostic timing in NP [1,5,10].

Within this context, the interval between clinical deterioration and CT confirmation in our cases reflects the real-world workflow of a resource-limited ICU rather than adherence to predefined, time-based diagnostic protocols. In all three patients, repeat chest CT was triggered by unexpected respiratory or systemic deterioration and failure to improve despite appropriate antimicrobial therapy and supportive care. In Cases 1 and 2, abrupt worsening of hypoxemia with progression to refractory ARDS prompted CT reassessment and informed subsequent escalation decisions, including transfer for ECMO evaluation. In Case 3, progressive multiorgan dysfunction, refractory shock, and metabolic derangement led to repeat imaging that confirmed advanced necrotizing disease but coincided with clinical trajectories ultimately precluding ECMO candidacy. These case-specific turning points illustrate how, in peripheral ICUs, imaging, escalation, and transfer decisions are closely linked to evolving clinical instability and institutional resources rather than to strict diagnostic timelines.

The spectrum of CT findings observed in our series, ranging from extensive bilateral cavitation in Case 1 to liquefactive parenchymal destruction in Case 3, further aligns with the recognized continuum between lung abscess, necrotizing pneumonia, and pulmonary gangrene described in the literature [4,11]. Framing these intervals as “time to radiological confirmation,” rather than diagnostic delay, may therefore better capture this dynamic and avoid implying a causal relationship between CT timing and outcomes, which remains controversial in NP.

When compared with contemporary adult NP cohorts such as the one studied by Boppana et al., our patients align with similar demographic and clinical profiles (middle-aged adults with limited baseline comorbidity, predominantly monomicrobial infections, and frequent need for mechanical ventilation and organ support) [1]. ECMO was used in one of three cases (33%), a proportion comparable to the 30% reported in recent severe NP cohorts, while overall mortality (1/3, 33%) was also consistent with published outcomes in critically ill, ventilated NP patients [1,10]. These parallels reinforce that the severity, microbiological patterns, and clinical trajectories observed in our cases are broadly aligned with current evidence [1,10].

All patients had high CURB-65 and PSI scores, indicating the need for ICU care and matching the severe clinical course that later developed [9,12,13]. The rapid onset of respiratory failure requiring invasive mechanical ventilation is consistent with the severe disease described in prior series [7]. Both Cases 1 and 2 progressed to refractory ARDS despite lung-protective ventilation and prone positioning, ultimately requiring transfer to a tertiary center for VV-ECMO evaluation, reflecting the accepted role of ECMO in fulminant NP [8]. This pattern mirrors findings by Boppana et al., in which mechanical ventilation was the strongest independent predictor of mortality [1]. In contrast, Case 3 deteriorated with severe multiorgan failure and was not considered eligible for ECMO rescue, reflecting the heterogeneity in clinical trajectories and candidacy for advanced support [8].

Multiorgan dysfunction was observed in all cases, although the severity varied. All patients developed acute kidney injury, and Cases 2 and 3 required vasopressor support for septic shock. Case 3 progressed to anuric renal failure, severe rhabdomyolysis, and refractory metabolic acidosis, necessitating CRRT with an HA380 hemoadsorption cartridge. Extracorporeal adsorption techniques are emerging adjuncts in hyperinflammatory states, although current evidence remains limited and largely experimental [14]. At our hospital, serum IL-6 measurement, typically used to guide hemoadsorption initiation, is unavailable; however, a marked rise in myoglobin during the critical period supported the decision to employ an HA380 hemoadsorption cartridge. Consequently, the use of HA380 in this patient should be interpreted as context-specific supportive therapy rather than an intervention with established efficacy. These severe systemic manifestations are consistent with the high mortality reported in NP, ranging from 20-40% in earlier series and 24.6% in a recent 2025 cohort [1,5]. The fatal outcome in Case 3 aligns with this expected mortality and underscores the prognostic impact of multiorgan failure.

Although medical therapy remains the cornerstone of management, surgical intervention may be required in patients who fail to improve or who develop complications such as massive hemoptysis, bronchopleural fistula with persistent pneumothorax, loculated empyema, or progression to pulmonary gangrene [5,6,15]. None of our patients required lung resection. Surgical intervention is relatively uncommon overall, but the recent tertiary-center experience reported by Boppana et al. shows that a notable subset of patients may ultimately require operative management, often through minimally invasive approaches such as video-assisted thoracoscopic surgery (VATS), highlighting how disease severity and institutional resources can influence the threshold for surgery [1]. Some studies have shown that the presence of necrosis does not necessarily correlate with increased mortality but is instead associated with longer hospitalization and a greater need for interventions such as pleural drainage [16].

Resource limitation in this peripheral hospital also played a role in shaping these patients’ clinical courses. Lack of immediate access to interventional radiology and the need for external ECMO transfer may have contributed to deterioration in Cases 1 and 3. These barriers have been described in Portuguese and international literature and remain a major determinant of outcomes in NP [2,9].

Point-of-care lung ultrasound (LUS) is increasingly recognized as a useful adjunct in suspected NP, particularly in ventilated or unstable patients. Recent adult series indicate that serial LUS can detect subpleural necrosis, microabscesses, and evolving cavitations, prompting earlier CT when transport is risky or imaging access is delayed [17-19]. Current Society of Critical Care Medicine critical care ultrasonography guidance likewise supports LUS as a bedside tool that can expedite diagnosis in settings with limited availability of advanced imaging [20].

When compared with other published case series, the clinical progression in our patients, with a rapid evolution from severe CAP to extensive necrosis within 5 to 11 days, aligns with the timelines typically associated with highly virulent pathogens [1,5,7]. The combination of bacteremia, rapidly worsening respiratory failure, and multiorgan involvement observed in all three cases reflects the aggressive course well documented in contemporary NP reports [3].

This case series has several important limitations. First, the small sample size and absence of a control group restrict the ability to draw generalizable conclusions regarding risk factors, optimal imaging timing, or management strategies for necrotizing pneumonia. Second, the retrospective design relies on documentation recorded during routine clinical care, which may introduce incomplete data capture and reporting bias. Third, as a single-center experience from a peripheral institution, the findings may not be applicable to centers with different resources, workflows, or access to advanced interventions such as ECMO or interventional radiology. Additionally, detailed ventilatory parameters, such as positive end-expiratory pressure (PEEP) and PaO₂/FiO₂ ratios, were not systematically recorded in our peripheral ICU, limiting direct comparisons with larger published NP cohorts. Finally, long-term outcomes, including pulmonary function and radiologic resolution, were not assessed, precluding evaluation of post-discharge sequelae.

Despite these limitations, this work was valuable in promoting multidisciplinary discussion within the medical team and raising awareness of the diagnostic and organizational challenges posed by NP in resource-limited settings.

## Conclusions

NP remains a rare but severe complication of community-acquired pneumonia, in which timely diagnosis and early recognition of clinical deterioration are essential to limit progression to further organ dysfunction. In this descriptive series, repeat imaging was not driven by isolated physiologic thresholds but by a reproducible clinical trajectory, characterized by persistent or worsening respiratory failure, lack of improvement despite appropriate antimicrobial therapy, escalating inflammatory markers, recurrent fever, and evolving multiorgan dysfunction. Chest CT ultimately proved decisive for confirming the diagnosis in all cases, highlighting the importance of sustained clinical vigilance and timely reassessment, particularly in resource-limited settings where access to advanced imaging may be constrained. This trajectory-based approach to reassessment may help clinicians recognize necrotizing pneumonia earlier and guide escalation decisions in similar contexts.

Given the limited sample size, retrospective design, and absence of long-term follow-up, these observations cannot support conclusions about optimal imaging timing, risk stratification strategies, or the effectiveness of specific therapeutic interventions such as ECMO referral pathways or hemoadsorption. Likewise, this single-center experience may not be generalizable to institutions with different resources or organizational structures. Within these constraints, the cases illustrate practical challenges encountered in a peripheral ICU, including time to chest CT, rapid clinical deterioration, and the need for multidisciplinary coordination when managing patients with suspected NP. Rather than proposing outcome-modifying strategies, this series aims primarily to raise awareness of early clinical and radiologic warning signs and to encourage timely reassessment when expected improvement does not occur. Despite the limitations, this work contributed to important internal multidisciplinary discussions and helped increase local awareness of the diagnostic and organizational hurdles posed by necrotizing pneumonia in resource-limited settings.
